# Partial Congenital Absence of The Pericardium: A Case Report

**DOI:** 10.21470/1678-9741-2018-0357

**Published:** 2019

**Authors:** Narjes Benameur, Younes Arous, Manel landolsi, Sarra Chenik, Nejmeddine Ben Abdallah, Tarek Kraiem

**Affiliations:** 1 University of Tunis El Manar, Higher Institute of Medical Technologies of Tunis, Laboratory of Biophysics and Medical Technologies, Tunis, Tunisia.; 2 Military Hospital of Instruction of Tunis, Tunis, Tunisia.; 3 University of Tunis El Manar, Faculty of Medicine of Tunis, Tunis, Tunisia.

**Keywords:** Pericardium, Cardiac Imaging Techniques, Congenital Heart Defects - Anatomy and Histology

## Abstract

The complete or the partial absence of pericardium is a rare congenital malformation for which the patients are commonly asymptomatic and the diagnosis is incidental. The absence of the left side of the pericardium is the most common anomaly that is reported in the literature while the complete absence of pericardium or the absence of the right side of the pericardium are uncommon and their criteria are still unrecognized given their rare occurrence in clinical practice. This paper aims to report a case of 19-year-old male with the congenital partial absence of both sides of the pericardium and to highlight the symptoms and the different cardiac imaging modalities used to confirm the diagnosis of this defect.

**Table:** 

Abbreviations, acronyms & symbols	
CHD	= Congenital heart disease
CT	= Computed tomography
ECG	= Electrocardiogram
MRI	= Magnetic Resonance Imaging
RVEDi	= Right end-diastolic volume index

## INTRODUCTION

**Fig. 1:**
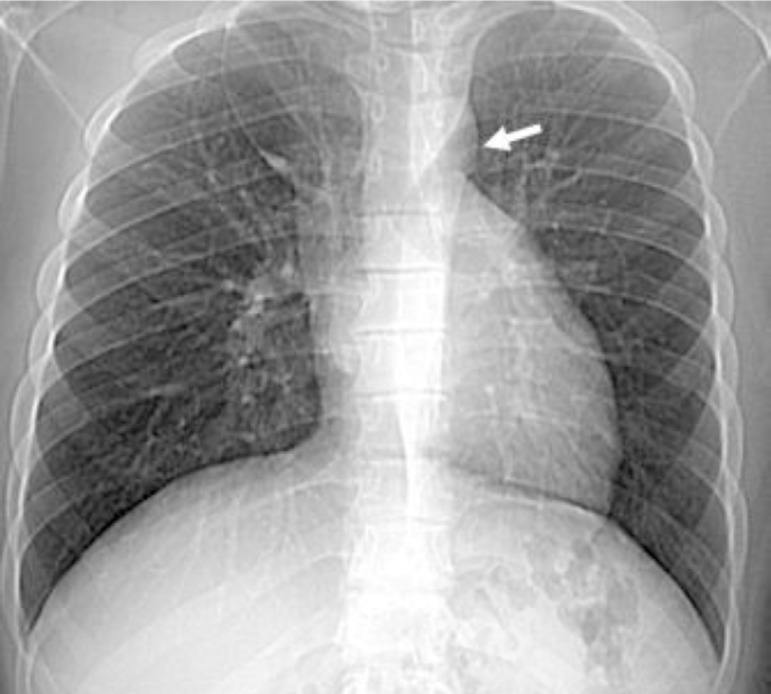
CT scout view was showing a lucent area between the aorta and pulmonary artery (white arrow).

**Fig. 2:**
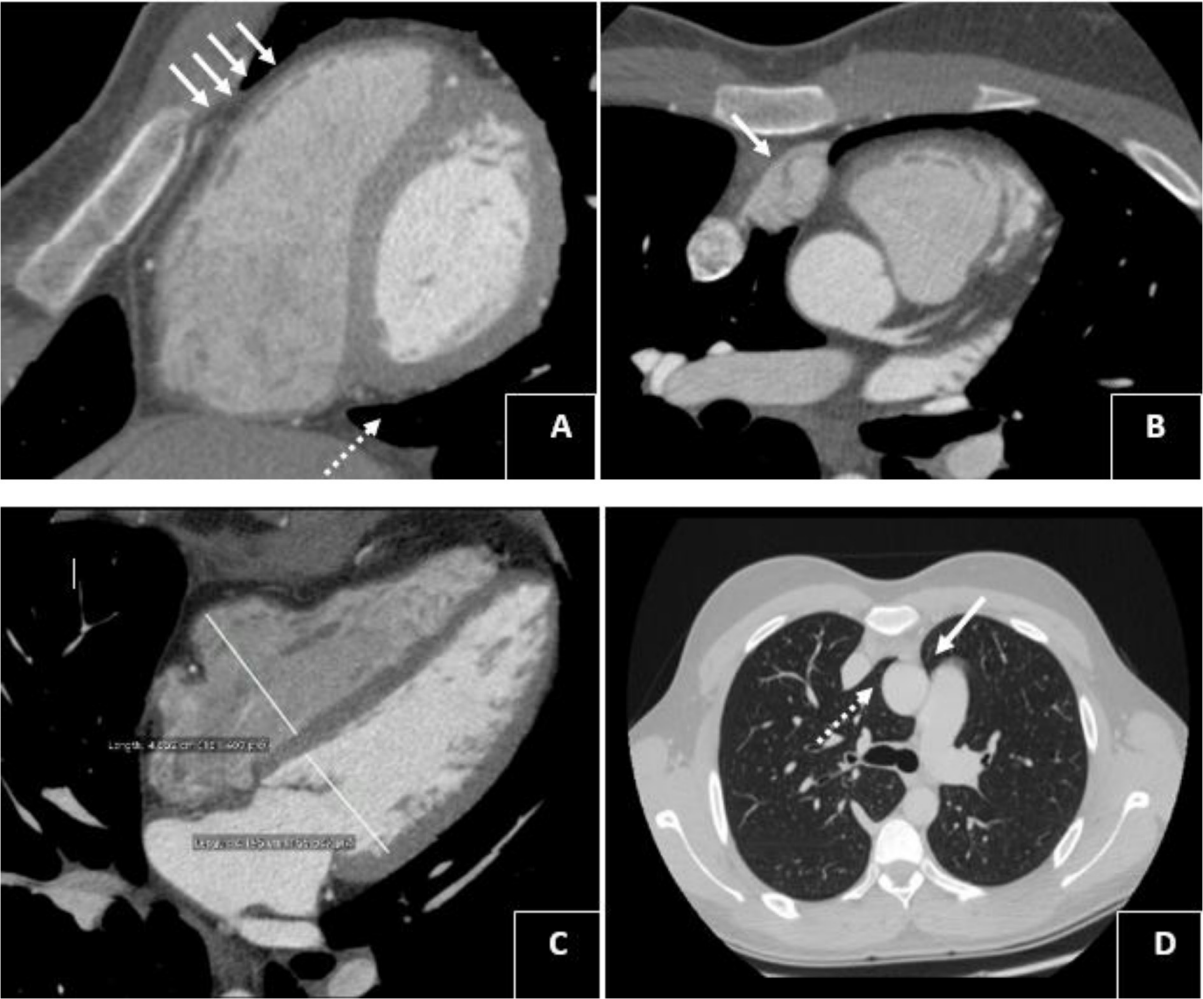
Cardiac CT demonstrating (A) the partial non-visualization of the right leaf (white arrows) and the interposition of the lung between the diaphragm and the base of the heart (dashed arrow), (B) right atrial appendage herniation (white arrow), (C) enlargement of right ventricular dimension (the right ventricular-to-left ventricular dimension ratio was 1.15), (D) interposition of the lung between the aorta and the superior vena cava (dashed arrow) and between the aorta and the pulmonary artery (white arrow).

A 19-year-old male patient was admitted to the radiology department of the Military Hospital of Tunis in July 2017 for a physical fitness test. His past medical history was not remarkable. He had no chest pain, no dyspnea or other specific signs. The electrocardiogram (ECG) indicated a normal sinus rhythm with a heart rate of 65 bpm and diffuse T wave inversion in anteroseptal leads. The transthoracic echocardiography and the transesophageal echocardiography showed an enlarged right ventricular dimension with no evidence of atrial septal defect. The Chest X-ray revealed a lucent area due to the interposition of lung tissue between the aorta and pulmonary artery (Figure 1). In addition, a cardiac computed tomography (CT) synchronized with ECG (64-slice detector, General Electric Medical Systems) was performed to exclude the presence of anomalous pulmonary venous return. The cardiac CT revealed a right atrial appendage herniation in the retrosternal space with a non-visualization of the superior portion of the right pericardium (Figure 2A and B).

Furthermore, the exam showed an interposition of lung tissue between the diaphragm and the base of the heart and between the aorta and the superior vena cava (Figure 2A and D) with no APVR found. It also confirmed the left lung interposition seen in the chest X-ray. The right ventricular-to-left ventricular dimension ratio computed by cardiac CT was 1.15 (Figure 2C). In order to exclude a possible occurrence of arrhythmogenic right ventricular dysplasia, a Magnetic Resonance Imaging (MRI) examination was performed.

Right end-diastolic volume index (RVEDi) and right ventricular ejection fraction obtained by MRI were respectively 87 ml/m^2^ and 58.6 % which is normal. All these findings revealed by cardiac imaging examinations confirmed a diagnosis of partial agenesis of right and left pericardium.

## DISCUSSION

The absence of pericardium is a rare congenital malformation generally characterized by non-specific symptoms. The majority of clinical cases reported in the literature showed that the congenital absence of pericardium (CAP) includes a total absence of pericardium and complete or partial absence of the left or the right side of the pericardium. The possible embryological origins of this pericardial anomaly are not well understood, but most studies demonstrated that it is due to defective development of the pleuropericardial membranes^[1]^. Among the different classes of CAP, the absence of the left side of the pericardium is the most common defect with a prevalence of 70 % while the incidence of the total absence of pericardium or the absence of the right side of the pericardium is still relatively uncommon^[2]^.

For this reason, the majority of literature reviews have focused on the possible symptoms, indications, and management algorithm that could be associated with the absence of the left side of the pericardium. Among the typical clinical signs, we could observe chest pain, dyspnea, the episode of acute respiratory distress leading to syncope, palpitation^[3]^. In the majority of reported cases of CAP, the clinical presentation is not specific. Patients are asymptomatic and the findings of this congenital disease are generally occurring incidentally. In this regard, the advent of different cardiac imaging modalities has significantly improved the specificity of the diagnosis of CAP by providing valuable information and indications that confirm the presence of this congenital heart disease.

In the current paper, a case of partial absence of right and left sides of the pericardium is presented. The findings of our study showed that the present case was asymptomatic without a remarkable medical history. In addition, the ECG showed a regular rhythm with diffuse T wave inversion in anteroseptal leads. While physical examination and ECG are not specific for the diagnosis of partial agenesis of the pericardium, the echocardiography contributes to the identification of several features related to this defect. The typical echocardiography findings of partial agenesis of left pericardium include cardiac levoposition, abnormal septal motion and increased mobility of the heart^[4]^.

A few case reports in the literature have shown the findings of echocardiography in patients with partial agenesis of the right pericardium. Among the echocardiography findings described by Shah et al^[1]^ associated with this type of defect, an enlarged right ventricle and hypertrophied right atrium with severe tricuspid regurgitation could be observed.

In the current case, the echocardiography showed an enlarged right ventricular dimension without an atrial septal defect. RVEDi obtained by MRI was 87 ml/m^2^ which is normal. This finding indicated that echocardiography showed a what appears to be a dilated right ventricle due to its anterior location. This would tend to yield a larger measured right ventricular dimension. As a result, the patient might be falsely labeled as affected by arrhythmogenic right ventricular dysplasia^[5]^.

In addition to the echocardiography, chest X-ray and cardiac CT play an essential role in the confirmation of the diagnosis and to the exclusion of some complications associated with partial agenesis of the pericardium. The chest X-ray showed that the interposition of lung tissue causes a lucent area between the aorta and pulmonary artery.

While echocardiography and chest X-ray exams are helpful in the extraction of some partial agenesis features as well as in the exclusion of other cardiac diseases, they are not able to confirm the diagnosis of partial absence of the pericardium. For this reason, a Chest CT or a Magnetic Resonance Imaging are always required in order to provide a definitive diagnosis and to assess the extent of the abnormality.

The chest CT features of the absence of right pericardium include visualizing herniation of right structure while the partial agenesis of the left pericardium is commonly revealed by the interposition of lung tissue between the aorta and pulmonary artery^[6]^. All These CT findings were shown in our case with a non-visualization of the superior portion of the right pericardium. Furthermore, the outcome of the cardiac CT showed an interposition of lung tissue between the aorta and the superior vena cava, which strongly confirms a diagnosis of partial agenesis of right and left pericardium.

The management of partial agenesis of right and left pericardium depends on the type and the extent of the pericardial defect. Usually, an intervention is not needed in case of patients with a complete absence of the pericardium. Complications are more occurring for patients with partial agenesis. Among the major complications that required a surgical intervention, we can note necrosis due to the herniation of the left atrial appendage, myocardial strangulation and incarceration of cardiac structures. In our case, the patient does not have these complications. Therefore, an intervention is not needed unless significant complications occur.

## CONCLUSION

In the current paper, we presented a case with the congenital partial absence of both sides of the pericardium. The outcomes of this study showed that this defect is usually asymptomatic. For this reason, a combination of several cardiac imaging modalities is needed to establish an accurate diagnosis of partial agenesis of right and left sides of the pericardium.

**Table:** 

Authors' roles & responsibilities	
YA	Substantial contributions to the conception or design of the work; or the acquisition, analysis, or interpretation of data for the work; final approval of the version to be published
ML	Substantial contributions to the conception or design of the work; or the acquisition, analysis, or interpretation of data for the work; final approval of the version to be published
SC	Substantial contributions to the conception or design of the work; or the acquisition, analysis, or interpretation of data for the work; final approval of the version to be published
TK	Substantial contributions to the conception or design of the work; or the acquisition, analysis, or interpretation of data for the work; final approval of the version to be published
